# Exit from quiescence displays a memory of cell growth and division

**DOI:** 10.1038/s41467-017-00367-0

**Published:** 2017-08-22

**Authors:** Xia Wang, Kotaro Fujimaki, Geoffrey C. Mitchell, Jungeun Sarah Kwon, Kimiko Della Croce, Chris Langsdorf, Hao Helen Zhang, Guang Yao

**Affiliations:** 1 Department of Molecular and Cellular Biology, University of Arizona, Tucson, AZ 85721 USA; 2 School of Biological and Medical Engineering, Hefei University of Technology, Hefei, Anhui 230009 China; 3 Department of Biology, Wofford College, Spartanburg, SC 29303 USA; 4 Molecular Probes, Thermo Fisher Scientific, Eugene, OR 97402 USA; 5 Department of Mathematics, University of Arizona, Tucson, AZ 85721 USA; 6 Arizona Cancer Centre, University of Arizona, Tucson, AZ 85719 USA

## Abstract

Reactivating quiescent cells to proliferate is critical to tissue repair and homoeostasis. Quiescence exit is highly noisy even for genetically identical cells under the same environmental conditions. Deregulation of quiescence exit is associated with many diseases, but cellular mechanisms underlying the noisy process of exiting quiescence are poorly understood. Here we show that the heterogeneity of quiescence exit reflects a memory of preceding cell growth at quiescence induction and immediate division history before quiescence entry, and that such a memory is reflected in cell size at a coarse scale. The deterministic memory effects of preceding cell cycle, coupled with the stochastic dynamics of an Rb-E2F bistable switch, jointly and quantitatively explain quiescence-exit heterogeneity. As such, quiescence can be defined as a distinct state outside of the cell cycle while displaying a sequential cell order reflecting preceding cell growth and division variations.

## Abstract (web summary)

The quiescence-exit process is noisy even in genetically identical cells under the same environmental conditions. Here the authors show that the heterogeneity of quiescence exit reflects a memory of preceding cell growth at quiescence induction and immediate division history prior to quiescence entry.

## Introduction

Out of the 10^13^ ~ 10^14^ cells in our body, the vast majority are non-dividing. While many non-dividing cells can no longer proliferate, such as cells in senescence or terminal differentiation, quiescent cells (e.g., lymphocytes, hepatocytes, stem and progenitor cells) retain their proliferative potential. In response to physiological signals, typically serum growth factors, quiescent cells can be activated to re-enter the cell cycle, which serves as the basis for tissue homoeostasis and repair^1–3^. Recent studies have shown that quiescence is not simply a passive fall-back state lacking proliferative activities, but is rather an actively maintained state^1, 2, 4^ that provides protection against long-term cellular stress and toxicity^1, 5^.

Quiescence exit is highly heterogeneous. In a clonal culture induced to quiescence by the same condition (e.g., serum starvation), individual quiescent cells exhibit significantly different paces in restarting the cell cycle upon serum stimulation^6–8^. Furthermore, upon non-saturating serum stimulation (at an intermediate concentration or with a short pulse), some cells re-enter the cell cycle while others remain quiescent^6, 7, 9^. Conceivably, a heterogeneous transition from quiescence to proliferation can be beneficial in vivo by avoiding exhausting a pool of quiescent cells completely with a single stimulus. It meanwhile poses a therapeutic challenge since cells remaining quiescent (e.g., certain cancer stem cells) are difficult to target. Mechanisms underlying the heterogeneity in quiescence exit are, however, poorly understood.

In this study, we set out to investigate what accounts for the heterogeneous quiescence exit in a supposedly homogeneous, clonal cell population under the same culture conditions. Particularly, is this heterogeneity caused by stochastic events, or deterministic and predictable variations, in the cell population? Given that a critical size control has been observed during the G1-S transition of cycling eukaryotic cells^10–12^, and that quiescent hematopoietic cells were shown to need to grow in size before restarting proliferation^13^, we first examined whether quiescence-exit heterogeneity was associated with cell size differences in a rat embryonic fibroblast (REF) cell model. We found that depending on experimental conditions, cell size may or may not appear to be associated with the observed quiescence-exit heterogeneity.

Further modelling and experimental analysis showed that quiescence-exit heterogeneity was associated with both the preceding cell growth at quiescence induction by serum starvation and the cell division status prior to quiescence entry (‘preceding cell growth and division’ for short). Meanwhile, cell size reflected preceding cell growth and division at a coarse but not fine scale. Our study showed that the deterministic variations in preceding cell cycle, coupled with stochastic noise in an Rb-E2F bistable switch that underlies the quiescence-to-proliferation transition^9, 14^, determine the heterogeneity of quiescence exit and cell cycle re-entry. Lastly, our analysis also suggests that quiescence, while being a distinct state outside of the cell cycle, displays a sequential cell order reflecting a memory of preceding cell growth and division. This new quiescence model helps settle the long debate over whether quiescent cells are located in a distinct G0 phase or simply paused along a G1 continuum, and reveals a previously underappreciated mechanism underlying the heterogeneous growth responses of quiescent cells.

## Results

### Quantify quiescence-exit heterogeneity of clonal cells

**Fig. 1:**
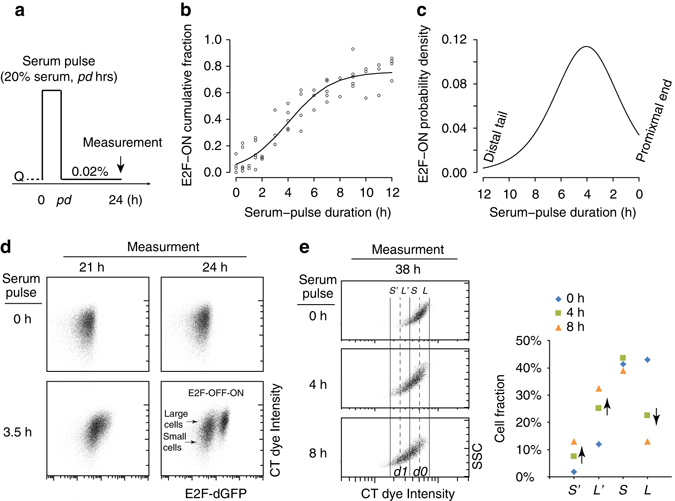
Quiescence-exit distribution and cell size correlation. **a** Experimental scheme for measuring quiescence-exit heterogeneity. Q, quiescent state (the same below). **b** Cumulative fraction of quiescence-exit (E2F-ON) cells over serum pulse duration. Each dot represents a unique sample. The sigmoid curve indicates a best fit of data points $\left( {y = \frac{a}{{1{\rm{ + }}{{\rm e}^{ - b(x - c)}}}}} \right)$. **c** Quiescence-exit distribution as the derivative of the sigmoidal curve in **b**. **d** Serum-starved quiescent REF/E23 cells were stained with CT violet dye at time 0, and stimulated with serum pulse at indicated durations. E2F-dGFP and CT levels were measured at the indicated time points after serum pulse initiation. **e** Serum-starved, CT-labelled quiescent cells as in **d** were stimulated with serum pulses at indicated durations. CT dye intensity and SSC were measured at the 38th hour after serum pulse initiation, as shown in the density plot (*left*); the percentage of cells in each labelled CT bin (*S’, L’, S, L*, as described in the text) is shown for each serum pulse group (*right*). For simplicity, CT bins were considered not to overlap. Cells of *d1* and *d0* labels correspond to those that divided and did not divide following a serum pulse, respectively

To better understand cellular mechanisms of quiescence-exit heterogeneity, we started by experimentally quantifying the profile of a clonal culture exiting quiescence. To this end, we first induced quiescence in isogeneic REFs (REF/E23 cells) by serum starvation (at 0.02% serum for 2 days, the same below unless otherwise noted). As shown in Supplementary Fig. 1a, serum starvation-induced quiescence was demonstrated by (i) the negative labelling of cells with a thymidine analogue, 5-ethynyl-2′-deoxyuridine (EdU), which is incorporated into the DNA of proliferating cells; (ii) a DNA profile of predominantly 2n but not 3–4n; and (iii) the E2F-OFF state of an Rb-E2F bistable switch (whose all-or-none activity correlates with cell proliferation and quiescence, respectively^9^), observed using a previously established E2F-dGFP reporter integrated in REF/E23 cells^9, 14^ (see Methods). Quiescent cells were stimulated with a serum pulse (at 20%, the same below unless otherwise noted; for pulse duration *pd* = 0–12 h) and subsequently returned to the serum starvation condition; the cell fraction that exited quiescence was measured at the 24th hour after serum pulse initiation (Fig. 1a). Quiescence exit was determined by the E2F-ON status (as in Supplementary Fig. 1b), which was consistent with the readout of the EdU incorporation assay (Supplementary Fig. 1c).

We observed that clonal REF/E23 cells exhibited significant heterogeneity in quiescence exit and cell cycle re-entry following serum stimulation: some cells responded to a serum pulse as short as 1–2 h; some did not respond until the serum pulse was over 8 h (Fig. 1b and Supplementary Table 1). When the serum pulse was sufficiently long (16–24 h), the E2F-ON cell fraction approached 1.0 (i.e., 100%, Supplementary Fig. 1d). Quantitatively, we found that the cumulative fraction of quiescence-exit cells (E2F-ON) could be fit with a sigmoidal curve over the serum pulse duration (Fig. 1b). Taking the derivative of this cumulative curve produced the probability density of E2F-ON cells at a given serum duration (Fig. 1c, note the flipped *x* axis). This probability distribution, which we called ‘quiescence-exit distribution’, exhibited a skewed normal-like form with a short tail at the right (proximal end) corresponding to cells readily responsive to transient serum pulses, and a long tail at the left (distal tail) corresponding to cells responsive only to long serum pulses.

### Larger cells exhibit higher quiescence-exit likelihood

We next investigated whether the quiescence-exit heterogeneity (Fig. 1b, c) was caused primarily by cellular stochasticity or certain deterministic variations in clonal cells. Given previous findings that S-phase entry is subject to a cell size control in yeast and likely certain types of mammalian cells^10–13^, we first examined whether cell size difference was correlated with quiescence-exit heterogeneity in REF/E23 fibroblasts. To this end, we labelled serum-starved quiescent cells right before serum stimulation (time 0) with a membrane permeable CellTrace (CT) Violet dye that binds covalently to cellular amines. The CT intensity is positively correlated with cell size measured electronically based on the Coulter principle (Supplementary Fig. 2a, b) as well as inferred based on the side-scatter and forward-scatter characteristics in flow cytometry^15^ (side-scatter and forward-scatter characteristics (SSC and FSC), respectively, Supplementary Fig. 2c, d). The CT dye is also stable and well-retained in cells for several days post stain^16^. Therefore, the CT intensity can serve to track the original sizes of quiescent cells after cell cycle re-entry. We found that following serum pulse stimulation (*pd* = 3.5 h), large quiescent cells (with high vs. low CT intensity) exhibited an apparently higher propensity to exit quiescence than small cells (switching from E2F-OFF to -ON, measured at the 24th hour after serum pulse initiation, Fig. 1d).

Consistently, at a later time point (the 38th hour after serum pulse initiation) that allowed cells to complete division following a serum pulse, a much higher percentage of large quiescent cells than small ones (CT intensity = *L* vs. *S*) exited quiescence and divided (*L*% dropped from 43.1% in the 0 h pulse group to 13.1% in the 8 h pulse group, compared to *S*% that dropped only slightly from 41.3% to 38.9%, Fig. 1e). Correspondingly, there was a much larger increase of *L* daughter cells (CT intensity = *L′* = *½L*) than *S* daughter cells (CT intensity = *S′* = *½S*, Fig. 1e). Put together, our data so far suggested that larger cells were more ready to exit quiescence in response to serum stimulation than smaller ones, consistent with the hypothesis that quiescence-exit likelihood is correlated with cell size.

### Cell size at quiescence induction governs quiescence exit

**Fig. 2:**
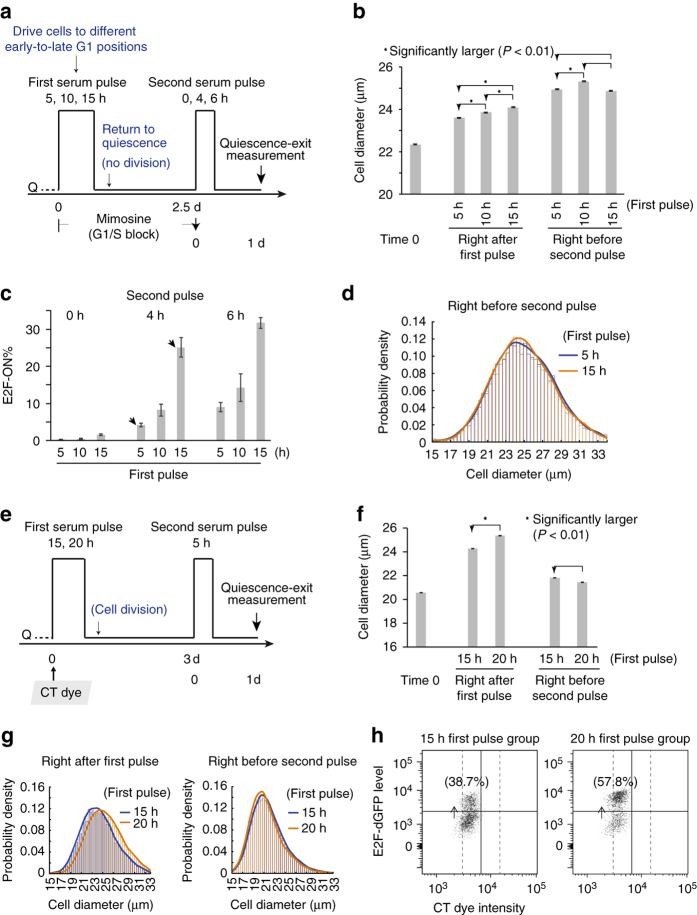
Quiescence-exit likelihood is correlated with cell size at quiescence induction. **a** Experimental scheme. E2F-dGFP activity was measured one day after the initiation of the second serum pulse. See text for details. **b** Cell size measured right before the first serum pulse (time 0), after the first pulse (of 5–15 h), and before the second serum pulse, following the scheme in **a**. *Star sign* indicates statistical significance *P* < 0.01) in one-sided (*arrow pointed*) *t*-test comparing the means of cell diameters in two cell populations (20,000–30,000 cells each). Error bar, s.e.m. **c** Quiescence-exit rate (E2F-ON%, mean ± s.e.m. of duplicates) in response to the second serum pulse for indicated durations as a function of the first serum pulse duration. **d** Probability density distribution of the cell size (diameter) of indicated cell samples in **b** right before the secnd serum pulse. The histogram curve represents a kernel smoothing fit of cell size distribution (the same below). **e** Experimental scheme. Quiescent cells were stained with CT violet dye at time 0, and E2F-dGFP activity was measured one day after the second pulse initiation. See text for details. **f** Cell size measured right before the first serum pulse (time 0), after the first pulse, and before the second pulse, following the scheme in **e**. The *star sign* and error bar are as in **b**. **g** Probability density distributions of cell size (diameter), of indicated cell samples in **f**. **h** Scatter plots of flow cytometry results. Percentages of E2F-ON cells following the 2nd pulse are shown. Each dot represents one single cell. Cells to the left of solid vertical line corresponded to divided cells following the first serum pulse (same gating as in Fig. 3c)

We next thought to further verify the correlation between cell size and quiescence exit in different experimental settings. Serum-starved quiescent cells were first stimulated with a serum pulse (first pulse, Fig. 2a) with an increasing duration (*pd* = 5, 10, or 15 h), and subsequently returned to quiescence by serum starvation (with G1-S progression blocked throughout this course by 0.4 mM mimosine, Fig. 2a). The first serum pulse with an increasing duration was purported to drive cells to progressively later G1 positions with increasing cell size, which was confirmed by actual size measurement based on the Coulter Principle (the same below; *right* after first pulse, Fig. 2b). Following serum starvation and with G1/S blockage, G1 cells withdrew from the cell cycle and returned to quiescence^14^, as confirmed by their low basal E2F-ON% and EdU labelling (0 h second pulse, Fig. 2c and Supplementary Fig. 3a). Surprisingly, after large and small cells at quiescence induction (following 15- vs. 5 h first pulse) returned to quiescence, they exhibited no significant difference in cell size, in both the mean and distribution (*right* before second pulse, Fig. 2b, d). The reason why larger cells at quiescence induction were not larger anymore after entering quiescence is not entirely clear (see Supplementary Discussion for possible reasons). That aside, larger cells at quiescence induction (e.g., following the 15- vs. 5 h first pulse) exhibited a significantly higher quiescence-exit percentage in response to the second serum pulse (e.g., 25.1% vs. 4.2% E2F-ON, arrow pointed, Fig. 2c, measured at the 24th hour after the initiation of the 4 h second pulse; for consistent parallel EdU assay readout, Supplementary Fig. 3a). That is, certain quiescence-exit heterogeneity was not correlated with the size of quiescent cells, but with the cell size at quiescence induction in the preceding cell cycle (*right* after first pulse, Fig. 2b).

Similar results were observed in a follow-up experiment, where cells were allowed to complete division (without mimosine blockage) before returning to quiescence. Specifically, as seen in Fig. 2e, serum-starved quiescent cells were first stimulated with a long serum pulse (*pd* = 15 or 20 h) that was sufficient to drive nearly all cells in the population to exit quiescence and divide (Supplementary Fig. 1d); cells were then returned to quiescence by serum starvation. Following a longer first serum pulse (20- vs. 15 h), cells progressed to a later cell cycle position with a significantly larger size (*right* after first pulse, Fig. 2f). These larger cells at quiescence induction were, however, not any larger after entering quiescence (*right* before second pulse, Fig. 2f, g, consistent with FSC/SSC measurements, Supplementary Fig. 3b), likely due to continuous growth of REF/E23 cell size under serum starvation (see Supplementary Discussion). Yet, larger cells at quiescence induction (following the 20- vs. 15 h first pulse) again exhibited a higher quiescence-exit percentage in response to the second serum pulse (57.8% vs. 38.7%, measured at the 24th hour after pulse initiation, Fig. 2h). In summary, results in both experiments in Fig. 2 showed that certain quiescence-exit heterogeneity was not correlated with the size of quiescent cells, but with the cell size at quiescence induction in the preceding cell cycle. That is, there should be another mechanism(s) controlling quiescence exit, beyond the size of quiescent cells.

### Immediate division history affects quiescence exit

Given that quiescence-exit likelihood is associated with preceding cell size at quiescence induction by serum starvation (Fig. 2), we speculated that there is a cellular property that reflects and ‘memorises’ serum-dependent cell cycle-coupled cell growth^17–19^. The increase of such serum-dependent property (‘*SDP*’ in short) couples with cell size increase in the presence of serum. After serum withdrawal, *SDP* accumulation essentially stops, so that *SDP* values reflect a snapshot of growth difference between cells at quiescence induction. Different from *SDP*, the size of REF/E23 cells continued to grow under serum starvation (compare ‘*right* after first pulse’ and ‘*right* before second pulse’ in Fig. 2b, also see Supplementary Fig. 4), which over time may blur the original size difference at quiescence induction (see Supplementary Discussion).

**Fig. 3:**
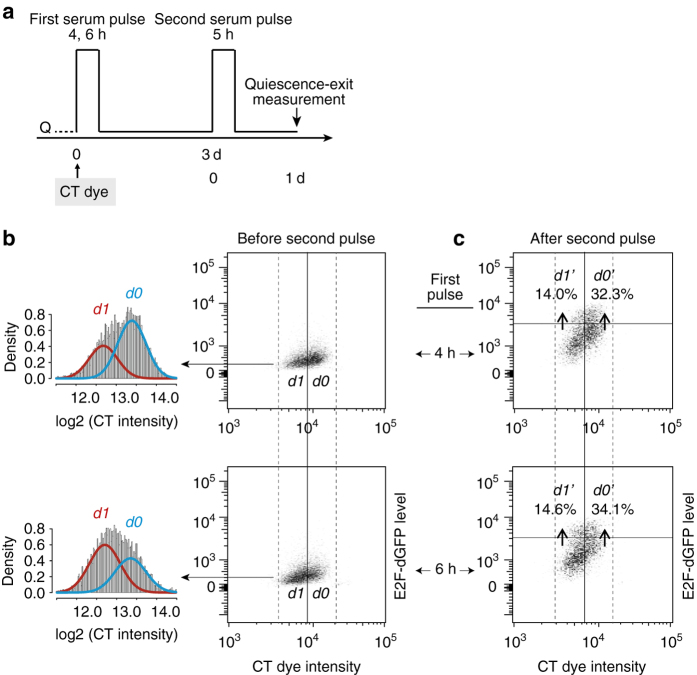
Recently divided cells are less likely to exit quiescence. **a** Experimental scheme. See text for details. **b** Non-divided (*d0*) and divided (*d1*) cells following the 1st serum pulse. (*left*) CT-intensity histograms binned in log2 scale and fitted with a two-component Gaussian mixture model, which was favoured over a one-component Gaussian model by showing a smaller Bayesian information criterion (BIC). (*right*) Density-plot regions with enriched *d1* and *d0* cells were separated according to their lower and higher CT dye intensity, respectively (*x* axis). The solid vertical line corresponds to the midpoint between the two modes of model-fitted *d1* and *d0* subpopulations (2^13.0^ = 8.2 × 10^3^). **c** Quiescence exit following the second serum pulse. Regions of *d1’* and *d0’* indicate subsets of *d1* and *d0* cells with E2F-ON status (above the horizontal gates), respectively. Percentages of *d1’* and *d0’* cells (over total *d1* and *d0* cells, respectively) are shown. Solid vertical and horizontal gating lines were placed through the least overlapped regions in smoothed density plots (Supplementary Fig. 5b). The slight left shift of the solid vertical gating line (compared to that in **b**) corresponded to dye decay over time

We considered that *SDP* conveyed to a quiescent cell affects its quiescence-exit likelihood in response to growth signals. We next determined how the *SDP* effect was transmitted through cell division. If *SDP* functions as freely diffusible factors, we would expect that following a short serum pulse (first pulse, Fig. 3a), cells that divided (*d1* cells) and did not divide (*d0* cells) before returning to quiescence exhibit similar quiescence-exit rates upon the second serum pulse (Fig. 3a; similar experimental scheme as the two-pulse experiments in Fig. 2, except that *d1* and *d0* cells have different immediate division history before the second pulse). This is because right after the first serum pulse, *SDP*_*d1*_ ≅ *SDP*_*d0*_, Size_*d1*_ ≅ Size_*d0*_; after division, *SDP*_*d1*_ ≅ *½ SDP*_*d0*_ and Size_*d1*_ ≅ *½* Size_*d0*_ (assuming similar size-change rate after serum starvation in both cell groups), therefore *SDP*_*d1*_/Size_*d1*_ ≅ *SDP*_*d0*_/Size_*d0*_ (i.e., similar *SDP* concentrations and thus similar serum responses in *d1* and *d0* cells). To test this prediction, we stimulated CT-dye labelled quiescent cells with the first serum pulse (*pd* = 4 or 6 h, Fig. 3a), which produced *d1* and *d0* cells with low and high CT intensity (and small and large size), respectively (Fig. 3b and Supplementary Fig. 5a). When stimulated with the second serum pulse (*pd* = 5 h), *d1* cells were found to exhibit a much smaller quiescence-exit rate (E2F-ON%) than *d0* cells (e.g., *d1′/d1* = 14.0% vs. *d0′/d0* = 32.3%, 4 h first pulse group; Fig. 3c), as measured at the 24th hour after the second pulse initiation. This result showed that the immediate division history prior to quiescence entry can greatly affect quiescence exit, so that recently divided cells were less likely to re-enter the cell cycle and divide again than non-divided cells. It also suggested that *SDP* may not correspond to stable factors that are freely diffusible; rather, it may be associated with, e.g., certain cell membrane-bound receptors or transporters, whose two-dimensional densities are reduced after three-dimensional cell division (see Discussion), leading to reduced serum responses.

### Model the control of quiescence-exit heterogeneity

**Fig. 4:**
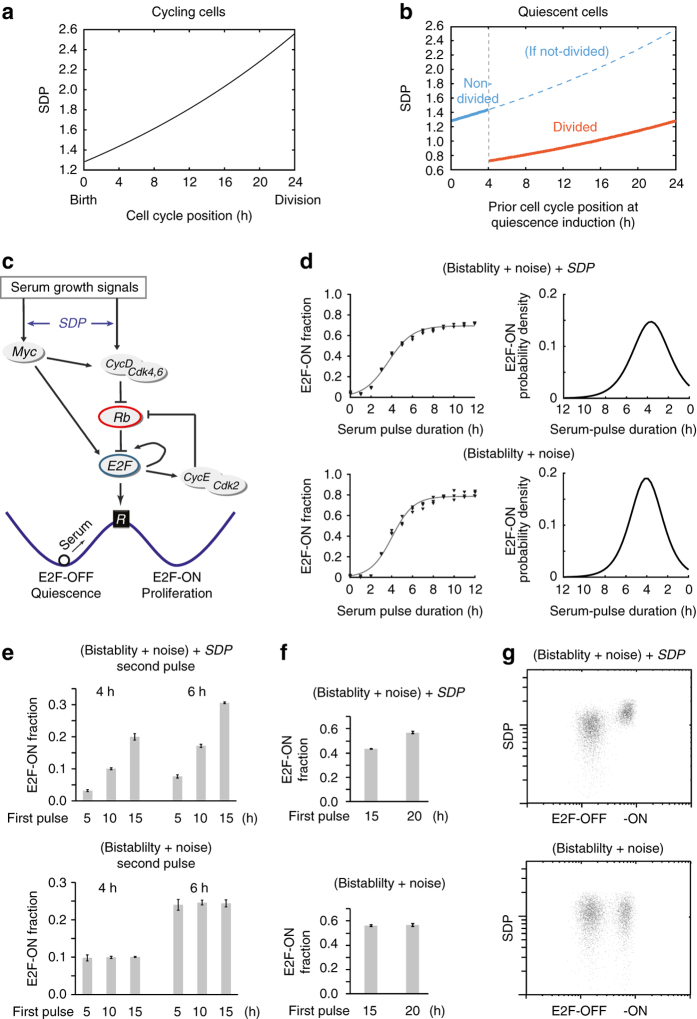
Model the memory effects of preceding cell growth and division on quiescence exit. **a** *SDP* accumulation in a cycling cell from cell birth to division, during which *SDP* value doubles. Cell cycle position refers to the hours after cell birth, in the presence of serum. **b** *SDP* distribution in a population of quiescent cells, related to preceding cell cycle positions at quiescence induction. Cells past the R-point (4 h after cell birth) enter quiescence after division with *SDP* reduced by half. **c** Rb-E2F bistable network that mediates the all-or-none transition from quiescence-to-proliferation. See text for details. **d**–**g** Simulation results based on the stochastic *SDP*-Rb-E2F model (*top*) vs. stochastic Rb-E2F model without *SDP* (*bottom*). **d** (*left*) Each data point represents the E2F-ON fraction calculated from 1000 simulations. Sigmoid curve indicates a best fit of triplicate data points (with the function form as in Fig. 1b). (*right*) Quiescence-exit distribution as the derivative of the sigmoidal curve on the left. **e**, **f** Each column represents the E2F-ON fraction (mean ± s.e.m.) calculated from three sets of stochastic simulations (500 runs each). Cells were simulated according to experimental schemes in Fig. 2a, e (with batch-variation factor *S*_*c*_ = 0.55 and 1.15 for the second pulse, respectively). **g** Results from 5000 simulated cells are shown according to experimental procedure in Fig. 1d (*pd* = 3.5 h). Each dot represents one stochastic simulation of a single cell. Each cell was randomly assigned a *SDP* value (in log-normal distribution, *y* axis), with or without (*top* and *bottom*, respectively) coupling to serum response elements in the Rb-E2F bistable model. The *x* axis indicates the simulated E2F molecule number at the 24th model hour after serum pulse initiation (plus autofluorescence assumed in log-normal distribution, mean = 100)

We next tried to model the control of quiescence-exit heterogeneity by preceding cell growth and division. First, we approximated *SDP* accumulation under serum stimulation in proliferating cells with an exponential function (Fig. 4a, similar to that of serum-dependent cell growth^20–23^). We also considered that (i) a cycling cell population is uniformly distributed along the cell cycle (considering other distribution forms did not change the qualitative model results, see Discussion); and (ii) cells past the R-point irreversibly commit to cell division^24^. The R-point was set in our model at the 4th hour after the initiation of serum stimulation, based on the mode of the quiescence-exit distribution (Fig. 1c), which was also consistent with previous observations in REF/E23 cells^9^. After serum is withdrawn, cells not reaching the R-point enter quiescence with *SDP* values corresponding to their then cell cycle positions, i.e., time duration under serum stimulation (solid blue segment, Fig. 4b), and cells past the R-point continue to divide before entering quiescence, with their accumulated *SDP* by the time of serum starvation split in half into two daughter cells (orange segment, Fig. 4b). Therefore, *SDP* in our model reflects a memory of preceding cell growth at quiescence induction and division history (referred collectively as ‘*SDP* memory’ for short).

To model the influence of *SDP* memory on quiescence exit, we coupled *SDP* with the Rb-E2F pathway that controls the quiescence-to-proliferation transition^25–29^. E2F activators (E2F1, 2 and 3a; here referred to as E2F for simplicity) are a family of transcription factors that is both necessary and sufficient for cell cycle entry^30, 31^. E2F is inhibited by Rb proteins, which are in turn inhibited by cyclin/Cdks (e.g., CycD/Cdk4,6 and CycE/Cdk2) via phosphorylation (Fig. 4c). Previously, we have modelled and experimentally shown that the Rb-E2F pathway functions as a bistable switch to convert transient and graded serum growth signals into an all-or-none E2F activation, which underlies the all-or-none quiescence exit and cell cycle entry upon serum stimulation^9, 32^. Here, we set *SDP* to affect the serum response elements (Myc and CycD synthesis) in the Rb-E2F network (Fig. 4c). For simplicity, we modelled the effect of *SDP* on serum response as proportional to the *SDP* value (considering other forms did not change qualitative model outcomes, see Discussion). The resultant Rb-E2F bistable switch model with a *SDP* memory (‘*SDP*-Rb-E2F model’ for short) is composed of seven coupled ordinary differential equations (Supplementary Table 2). In its stochastic version (see Methods), cell-to-cell variations in a population of quiescent cells are caused by both the Rb-E2F system noise (a stochastic component) and the *SDP* memory (a deterministic component) of individual cells.

Simulation results from the *SDP*-Rb-E2F model were consistent with observations in both sets of experimental conditions in Figs 1 and 2. First, we found that the experimentally observed sigmoidal cumulative quiescence-exit curve (Fig. 1b) and accordingly the skewed normal-like quiescence-exit distribution (Fig. 1c) can be generated from our previous Rb-E2F bistable switch model, regardless of considering *SDP* or not (Fig. 4d). This result is consistent with the threshold-linear property of bistable systems as previously noted^33^, and suggests that a noisy Rb-E2F bistable switch can explain a part of quiescence-exit heterogeneity.

The *SDP* memory was necessary for reproducing the dependence of quiescence-exit variations on preceding cell growth and division. With the same immediate division history (within either the blue or orange segment, Fig. 4b), quiescent cells previously at later cell cycle positions had larger *SDP* than those at earlier positions, making them more responsive to serum signals to turn on the Rb-E2F switch. Consistently, simulation results (top panel of Fig. 4e, f) were consistent with previous experimental results (Fig. 2c, h, where cells were all without and with cell division, respectively, before entering quiescence after the first serum pulse). With different immediate division history before quiescence entry, recently divided cells (orange segment, Fig. 4b) had smaller *SDP* than non-divided ones (solid blue segment, Fig. 4b), making them less responsive to serum signals. Consistently, simulation results (Fig. 4g) reproduced earlier experimental observations (Fig. 1d, where recently divided and non-divided cells were produced following serum starvation of asynchronously growing cells, depending on the relative cell positions to the R-point). Without considering the *SDP* memory, variations in quiescence-exit rate were caused only by noise in the Rb-E2F bistable system, uncorrelated with preceding cell growth and division (*bottom* panels, Fig. 4e–g).

### Quiescence displays a memory of preceding growth and division

We realized that our results above suggested a new model for cellular quiescence. The nature of the quiescent state has been the subject of a long debate^34^. Two competing models considered that quiescent cells are either located in a distinct G0 phase outside of the cell cycle^7, 35, 36^ (G0 model), or paused sequentially along a G1 continuum^37–39^ (G1 model). Correspondingly, quiescence exit is probabilistic according to the G0 model^40–43^, but deterministic according to the G1 model (following the sequential G1 order)^37–39^. Our findings instead suggested a hybrid model: quiescence exit is determined jointly by a stochastic (Rb-E2F system noise) and a deterministic (*SDP* memory) component.

**Fig. 5:**
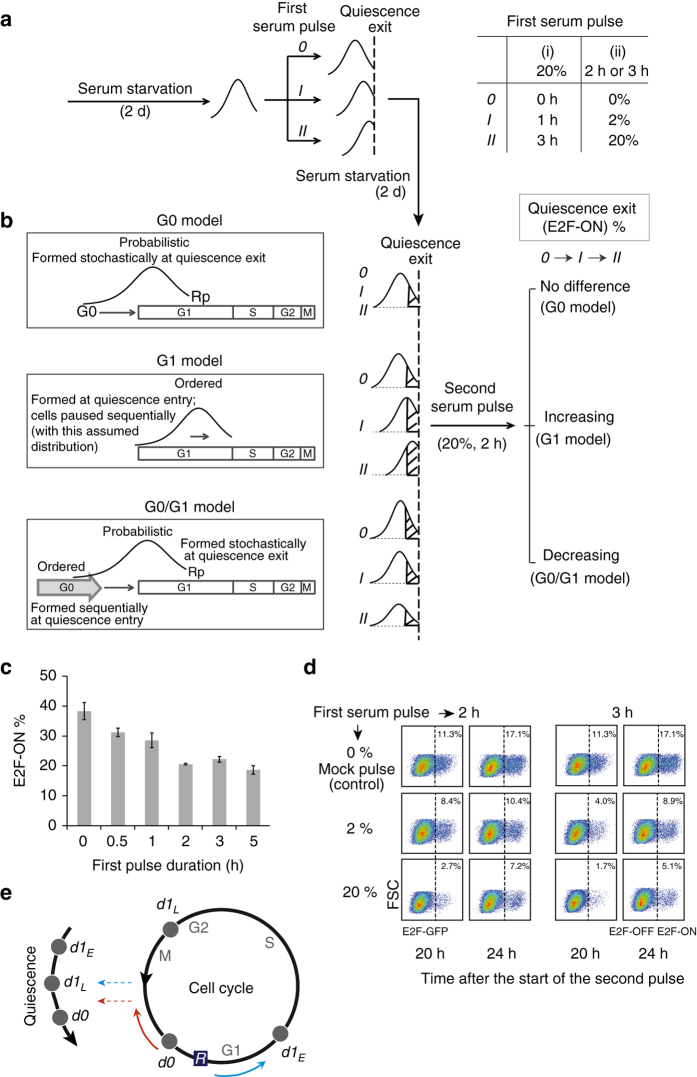
Experimental tests of three competing quiescence models. **a**, **b** Experimental schemes. See text for details. Two sets of first serum pulse with increasing duration (i) or concentration (ii) are shown on the right of **a**. Three competing models (G0, G1 and G0/G1) for quiescence, with their explanations of quiescence-exit heterogeneity (probabilistic, ordered and both, respectively) are shown on the *left* in **b**. *Rp*, restriction point. **c**, **d** Following experimental schemes in **a** and **b**, after the first serum pulse of increasing durations (0–5 h at 20%, **c**) or increasing concentrations (0–20% for either 2 or 3 h, **d**), cells were returned to quiescence by serum starvation, and then subject to the second serum pulse. Percentages of E2F-ON cells were measured at the 24th hour (**c**, mean ± s.e.m. of triplicates) or at the 20th and 24th hour **d** after the initiation of the second serum pulse. **e** The new quiescence model. Quiescent cells are located in the G0 state outside of the cell cycle while arrayed in a sequence reflecting their preceding cell growth and division, which together with noise in the Rb-E2F bistable system, determines quiescence-exit likelihood (*d0*: non-divided cells, *d1*_L_ *and d1*_E_: divided cells previously at late and early cell cycle positions at quiescence induction, respectively)

We next experimentally tested the three quiescence models. To this end, we stimulated serum-starved quiescent cells with the first serum pulse with varying strength or duration (Fig. 5a, *right*). Cells were then returned to quiescence under serum starvation for 2 days, and subsequently stimulated again with the second serum pulse (at 20% for 2 h). Regardless of the nature of the quiescence-exit distribution (probabilistic, ordered, or both; Fig. 5b, *left*), following a stronger first pulse (*II* > *I* > *0*; Fig. 5a, *right*), more cells from the proximal distribution end (i.e., on the *right*) exited quiescence, shifting the distribution peak closer to the quiescence-exit boundary (Fig. 5a, *left*). In the G1 model, this new distribution will remain, resulting in a larger cell fraction exiting quiescence following the second serum pulse (i.e., ‘increasing’ in the case order of *0, I, II*, see the shaded distribution fractions, Fig. 5b; see Supplementary Fig. 6a for the actual calculated fractions considering the divided cells following the first pulse). In the G0 model, since the quiescence-exit distribution is formed stochastically and temporarily (i.e., reset each time), the cell fraction exiting quiescence following the second serum pulse would be independent of the first pulse variations (i.e., ‘no difference’ in the case order of *0, I, II*, Fig. 5b). In contrast, our hybrid G0/G1 model predicted a ‘decreasing’ trend in the case order of *0, I, II* (Fig. 5b), as a stronger first serum pulse (*II > I > 0*) drives more cells to divide without full growth, leading to reduced *SDP* and quiescence-exit likelihood (see Supplementary Fig. 6b–d for simulation results and explanation in detail). Note that this case is different from Fig. 2a–d in which cell division was blocked and thus a longer first serum pulse led to increasing *SDP* and quiescence-exit likelihood. Also note that when the first serum pulse is long (>7 h, Supplementary Fig. 6e), an increasing pulse duration results in increasing quiescence-exit likelihood, which is consistent with the trend in Fig. 2e–h. This is because when the serum pulse is over 7 h, the increased rate of cell division (Fig. 1b) is much smaller than that of *SDP* accumulation (Fig. 4a).

As seen in Fig. 5c, our experimental results showed that after cells were exposed to an increasingly longer first serum pulse (at 20% for 0–5 h), they indeed had neither an increasing nor a constant, but a decreasing cell fraction that exited quiescence following the second serum pulse (significant negative slope, *P* < 0.05). Similarly, after quiescent cells were exposed to the first serum pulse with an increasingly higher serum concentration (0, 2%, 20%, for either 2 or 3 h), the cell fraction that exited quiescence in response to the second serum pulse was neither increasing nor constant, but decreasing, as indicated by the E2F-ON% measured at both the 20th and 24th hour after the initiation of the second pulse (Fig. 5d). These experimental observations fit well the hybrid G0/G1 quiescence model, but not the G0 or G1 model.

## Discussion

In this study, our experimental and modelling results suggest that quiescent cells stay at a distinct G0 phase outside of the cell cycle while displaying a memory of their preceding cell growth and division (Fig. 5e). The *SDP* memory affects the response of the Rb-E2F bistable switch in individual quiescent cells to serum signals. Correspondingly, in a cell population induced to quiescence by serum starvation, recently divided cells (*d1*) are less likely to respond to the next serum stimulation and exit quiescence than non-divided cells (*d0*). In each of the *d1* and *d0* subpopulations, those at quiescence induction progressed to later positions in the preceding cell cycle (with further accumulated *SDP*) are more likely to exit quiescence in response to the next serum stimulation than those previously at earlier positions (Fig. 5e).

Our work shows that the decisions of individual quiescent cells to exit quiescence upon serum stimulation are jointly determined by the deterministic *SDP* memory (reflecting the preceding cell growth and division) and stochastic noise in the Rb-E2F bistable switch. Our model simulations suggest that both factors are critical in this regard, as control models that consider either the *SDP* memory or Rb-E2F system noise alone cannot reproduce experimental results (Supplementary Fig. 7c, d). The behaviours of our model are robust otherwise and do not depend on particular formulations such as whether cells follow a uniform or normal distribution (with varying mode positions) along the cell cycle, or whether the cell cycle duration is 24 or 16 h (Supplementary Fig. 7b). The *SDP*-Rb-E2F model helps explain the heterogeneous growth responses of quiescent cells, as well as helps settle the long debate over the location of the quiescent state.

*SDP* is positively, but not perfectly, correlated with cell size. The continuous size growth of REF/E23 cells under serum starvation (Supplementary Fig. 4) is consistent with earlier findings that quiescent fibroblasts exhibit high metabolic activity^44^; it meanwhile contributes to the decoupling between cell size and *SDP* (even without considering variations introduced along cell size changes over time, see Supplementary Discussion). When the cell difference in preceding growth/division is large (e.g., in Fig. 1d, e, when an asynchronous culture was induced to quiescence), *SDP* difference is correspondingly large (i.e., between solid blue and orange segments, Fig. 4b), which can be approximated by cell size difference. In comparison, when the cell difference in preceding growth/division is small (e.g., in Fig. 2, with a difference of several hours in the first pulse duration and no difference in division status afterwards), *SDP* difference is correspondingly small (i.e., within either the blue or orange segment, Fig. 4b), which cannot be accurately reflected by cell size difference. On the other hand, if cell size continues to decrease after serum starvation, it will likely also decouple from *SDP*; if cell size drops below a certain threshold as seen in hematopoietic cells^13^, cells may be subject to a *SDP*-independent size control. We note this is not the case in REF/E23 cells, whose size continues to grow under serum starvation – without division, they become even larger than their proliferating counterparts (Supplementary Fig. 4). In short, cell size is associated with *SDP* at a coarse but not fine scale, which explains the puzzling observations regarding the correlation between cell size and quiescence exit depending on the scale of cell differences measured (Figs 1d, e and 2).

The exact nature of the *SDP* memory remains a significant unanswered question. Our work suggests that *SDP* accumulates in a serum-dependent manner, which is relatively stable and once conveyed to quiescent cells, affects the cell response to incoming growth signals. Meanwhile, the effective *SDP* concentration drops after cell division, so that recently divided cells are less likely to re-enter the cell cycle than non-divided cells. A plausible example that fulfils the above criteria might be certain cell membrane-bound growth factor receptors or nutrient transporters, whose accumulations are coupled with cell volume increase in the presence of serum. As a result, the *SDP* membrane intensity *SDP*_mi_ (responsible for promoting cell growth) is proportional to cell radius $r\left( {SD{P_{mi}} = \frac{\rm{amount}}{\rm{area}} \propto \frac{{{r^3}}}{{{r^2}}} = r} \right)$. Cells at late versus early cell cycle positions at quiescence induction have larger *r* and thus larger *SDP*_mi_. After cell division, cell radius *r* is reduced and so is *SDP*_mi_ (put in another way, after division, *SDP* amount ∝ *r* ^3^ is reduced by 50% while membrane area ∝ *r* ^2^ is reduced less, by 37%, and thus *SDP*_mi_ decreases). For simplicity, here we modelled the effect of *SDP*_mi_ on the serum response of a cell as proportional to its *SDP* value (Supplementary Table 2). Considering other forms, e.g., proportional to the square or cube root of *SDP*, did not change qualitative model outcomes (Supplementary Fig. 7b). We note that there might be alternative *SDP* mechanisms. For example, it is possible that *SDP* is associated with certain cell cycle-promoting activity which increases in a serum-dependent manner; such activity is reduced (or the activity of its inhibitor is increased) in cells having undergone division (due to downstream negative regulations), leading to smaller *SDP* activity concentration in divided cells. Along this line, it has been shown recently that CDK2 activity increases monotonically along the cell cycle and bifurcates at mitosis into CDK2^inc^ and CDK2^low^, which drives corresponding cells to continue the next cell cycle or enter a G0-like quiescent state^45^. The CDK2 bifurcation is controlled by a CDK inhibitor p21^45^, which has been shown to regulate the population heterogeneity of quiescent cells^46^. CDK2-p21 is an important node in the Rb-E2F network (Fig. 4c). It needs to be further examined whether the *SDP* memory can be associated with CDK2-p21 or other nodes in the Rb-E2F network and its interacting pathways (including some cell ‘sizer’ proteins^18, 19^). Identifying the molecular nature of *SDP* memory in future investigations, and its generality across different cell types, will help better illustrate cellular mechanisms underlying the heterogeneous quiescence exit of normal and diseased cells.

## Methods

### Cell culture, quiescence induction and serum pulses

REF/E23 cells containing a stably integrated *E2F1* promoter-driven destabilised EGFP (E2F-dGFP) reporter were previously derived as a single-cell clone from REF52 cells^9^. Cells were maintained in Dulbecco’s Modified Eagle’s Medium (DMEM) (No. 31053, Gibco, Thermo Fisher) supplemented with 10% bovine growth serum (BGS, No. SH30541, Hyclone, GE Healthcare). Cells were regularly passed at a sub-confluent level. For quiescence entry, growing cells were trypsinized and seeded at ~10^5^ cells per well in six-well cell culture plates (No. 353046, Corning Falcon), washed twice with DMEM after cell attachment, and cultured in 0.02% BGS (serum-starvation medium) for 2 days. For a serum pulse treatment, serum-starvation medium was replaced with DMEM containing BGS at the indicated concentration and duration. After a serum pulse, cells were washed twice with DMEM and put back into serum-starvation medium.

### Assays for E2F activity, DNA content and EdU incorporation

To measure E2F activity in individual cells, REF/E23 cells were collected at indicated time points by trypsinization, fixed with 1% formaldehyde in Dulbecco’s phosphate-buffered saline (DPBS), and measured for fluorescence intensity of the E2F-GFP reporter. To measure DNA content, cells were trypsinized, spun down and resuspended in Nuclear Isolation Medium (0.5% bovine serum albumin and 0.1% NP-40 in phosphate-buffered saline) with propidium iodide (PI, 5 µg/ml) and 1% RNase A (R5000, Sigma), and measured for PI fluorescence intensity in individual cells. For EdU incorporation assay, EdU (1 μM) was added to culture medium at the indicated time and kept in the medium until cell harvest, when cells were trypsinized at indicated time points and subjected to a Click-iT EdU assay according to the manufacturer’s protocol (No. C10418, Life Technologies, Thermo Fisher). Flow cytometry was used to measure GFP, EdU, and PI signal intensity in individual cells. Approximately 5,000–15,000 cells from each sample were measured using a flow cytometer (LSR II from BD Biosciences, or Attune from AppliedBiosystems). Data were analysed using FlowJo software (v. 10.0).

### Cell labelling with tracking dye

Prior to the first serum pulse in a two-pulse experiment, quiescent cells when noted were stained with a cell tracking dye (CellTrace Violet) according to the manufacturer’s protocol (No. C34557, Molecular Probes, Thermo Fisher). Briefly, quiescent cells were washed with DPBS and incubated with 2 µM CellTrace Violet in DPBS, at 37°C for 20 min. Cells were then washed once with serum-free medium, followed by serum pulse stimulation as indicated in the text. Cells were harvested at indicated time points and the CellTrace (CT) fluorescence intensity of individual cells was measured by flow cytometry.

### Cell size measurement

Cell size was measured electronically using Moxi^TM^ Z Mini Automated Cell Counter (MXZ001, ORFLO) according to the manufacture’s protocol. Briefly, cells were harvested by trypsinization, centrifuged and resuspended in DPBS. The cell suspension was applied to Cell Count Cassettes Type M (MXC001, ORFLO), which was inserted into Moxi Z Cell Counter for cell size measurement. The measurement is based on the Coulter Principle, where changes in electrical impedance are proportional to the volume of nonconductive particles (e.g., cells suspended in an electrolyte) passing through an aperture in the device.

### Model development and SDP calculation

In the *SDP*-Rb-E2F model (Supplementary Table 2), the *SDP* element is coupled to the serum response elements (Myc and CycD synthesis) in our previously developed Rb-E2F bistable switch model^9^. For a cell *j* in a cycling population, its *SDP* value (*SDP*_*cj*_) is determined by its cell cycle position (*p*_*j*_): $SD{P_{cj}} = SD{P_{c0}} \cdot {2^{{p_j}/k}}$, where *SDP*_*c0*_ = 1.3 is the *SDP* value at cell birth, *k* is the cell cycle duration (hours), and *p*_*j*_∈(0,*k*) (Fig. 4a). When a cycling cell is induced to quiescence by serum starvation, if *p*_*j*_ <  4 (the R-point), the cell will withdraw from the cell cycle and enter quiescence, with the *SDP* value in quiescent cell, *SDP*_*j*_ = *SDP*_*cj*_; otherwise the cell will complete the division before entering quiescence, and accordingly the *SDP* value in two daughter cells $SD{P_j}{\rm{ = }}\frac{{SD{P_{cj}}}}{2}$ (Fig. 4b).

When a quiescent cell is stimulated with the first serum pulse (at concentration *C* for duration *D*), the cell gains and updates its *SDP* accordingly, $SD{P_j} = SD{P_j} \cdot {2^{{S_p}}} \cdot dv$, with ${S_p} = \frac{D}{k} \cdot \frac{{{C^2}}}{{{n^2} + {C^2}}}\left( {n = 4} \right)$ corresponding to the power of serum-dependent exponential *SDP* accumulation (Fig. 4a), *dv* = 0.5 if the cell divides (determined by simulated E2F ON/OFF status, see stochastic simulation) before returning to quiescence and *dv* = 1 otherwise. See Supplementary Fig. 6b for examples of *SDP* update in a cell population after a serum pulse. The updated *SDP* distribution is applied to simulate the response of the cell population to the second serum pulse.

### Stochastic simulation

Similar to ref. ^47^, we developed a stochastic differential equation (SDE) model based upon the ordinary differential equation (ODE) framework described in Supplementary Table 2. Specifically, we adopted the chemical Langevin formulation^48^:

$${X_i}\left( {t + {{\tau }}} \right) = {X_i}\left( t \right) + \mathop {\sum}\limits_{j = 1}^M {{v_{ji}}{a_j}\left[ {X\left( t \right)} \right]} {{\tau + }}\theta \mathop {\sum}\limits_{j = 1}^M {{v_{ji}}{{\left( {{a_j}\left[ {X\left( t \right)} \right]{{\tau }}} \right)}^{\frac{1}{2}}}\gamma + \delta \omega {{{\tau }}^{\frac{1}{2}}}} $$

where *X*_i_(*t*) denotes the molecule number of species *i*(*i* = 1,…,*n*) at time *t*, and $X\left( t \right) = \left( {{X_1}\left( t \right), \ldots ,{X_n}\left( t \right)} \right)$ is the system state at time *t*. Therefore, the temporal evolution of the system is measured based on the rates *a*_*j*_[*X*(*t*)] (*j* = 1,…,*M*) with the corresponding change of molecule number *i* described in *v*_*ji*_. Factors *γ* and *ω* are temporally uncorrelated, statistically independent normal Gaussian noises. In this equation, the first two terms represent deterministic kinetics, the third and fourth terms represent intrinsic and extrinsic noise. *θ* and *δ* are scaling factors to adjust the levels of intrinsic and extrinsic noise, respectively (*θ* = 0.45,*δ* = 20). Units of model parameters and species concentrations in the ODE model (Supplementary Tables 2 and 3) were converted to molecule numbers.

The simulated mean molecule numbers for E2F–ON and –OFF steady states were well separated, at ~500 and 20, respectively (at low-serum concentration = 0.3 following a serum pulse). We considered that when the mean E2F molecule number between the 30th and 50th model hour after the start of a serum pulse was over a cutoff value of 300, the cell re-enters the cell cycle with E2F-ON state. All SDEs were implemented and solved in Matlab.

### Data availability

The authors declare that all data supporting the findings of this study are available within the paper and its Supplementary Information file and also from the corresponding author upon reasonable request.

## Electronic supplementary material

Supplementary Information
