# Supplementary material for: Exit from quiescence displays a memory of cell growth and division
Source: Nat Commun. 2017 Aug 22;8:321. doi: 10.1038/s41467-017-00367-0 (PMC5567331; doi:10.1038/s41467-017-00367-0)
Supplement: Supplementary file 1 — Supplementary Information [file 41467_2017_367_MOESM1_ESM.pdf]

Title: Supplementary Information

Description: Supplementary Figures, Supplementary Tables, Supplementary Discussion, and Supplementary References

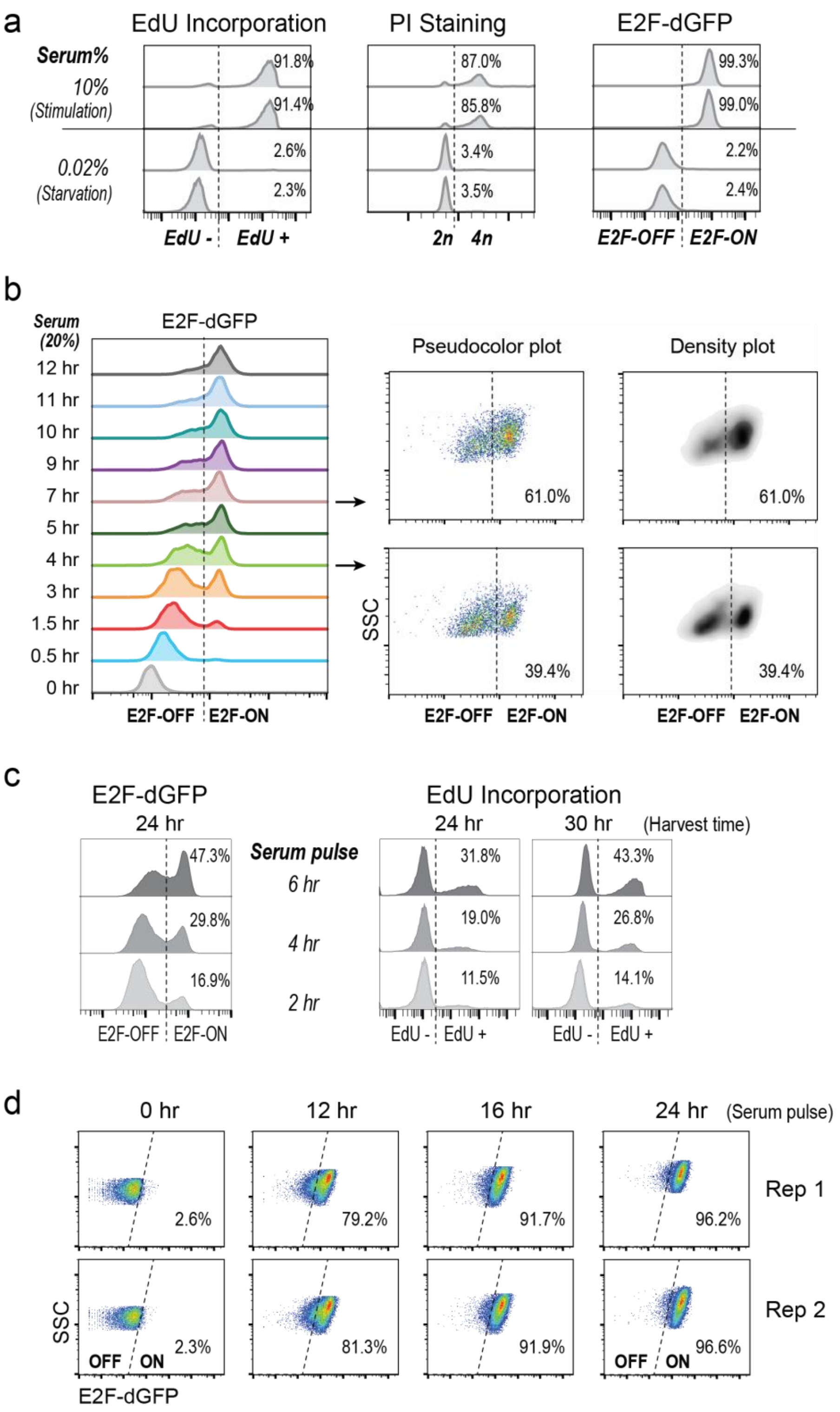

Supplementary Figure 1. REF/E23 cell model and E2F reporter for studying quiescence. (**a**) Quiescent vs. proliferating state determination. After 2-day serum starvation (0.02% serum), a portion of cells were harvested for PI and E2F-dGFP assays (see Methods), and remaining cells were cultured with EdU for one extra day before subject to EdU assay (to detect non-quiescent

cells with positive EdU incorporation). Also shown are control cells (top panel) that after serum starvation were stimulated with 10% serum for one day before subject to the above assays (EdU was included with 10% serum in EdU assay). The percentages of cells (duplicates) with EdU+, 3-4n DNA, and E2F-ON status, respectively, are shown. EdU assay appears more accurate than PI staining to measure the percentage of quiescence-exit cells, as PI assay does not distinguish cells remaining quiescent from those that re-enter the cell cycle and then divide (both with 2n DNA), while quiescence-exit cells all become EdU+ (which is easily distinguishable from EdU- even after cell division). The EdU+% was slightly lower than E2F-ON% in the 10% serum stimulation control, due to the delay between E2F turning ON and EdU incorporation (see **c**). (**b**) E2F-ON determination. Cells were treated as in Fig. 1a. E2F-ON and -OFF subpopulations were separated by a line going through the least overlapped region in between in the 2-dimensional pseudocolor or density plot (SSC vs. E2F-dGFP, see inset). (**c**) Comparison between E2F-dGFP and EdU assay readouts. Serum-starved quiescent cells were treated with serum pulses at indicated durations, and harvested at indicated time points after the initiation of serum pulse. In EdU assay, EdU was added to the culture medium right before the serum pulse and kept in the medium until cell harvest. Measured percentages of E2F-ON cells were positively correlated with those of EdU+ (which had a ~6-hr delay after E2F-ON as seen by comparing EdU+% at 30 hr and E2F-ON% at 24 hr). (**d**) E2F-ON cell fraction in response to long serum pulses. Cells were treated as in Fig. 1a with serum pulses at indicated durations. The E2F-ON% of cells (duplicates, measured at the 24th hr after the initiation of serum pulses) are shown.

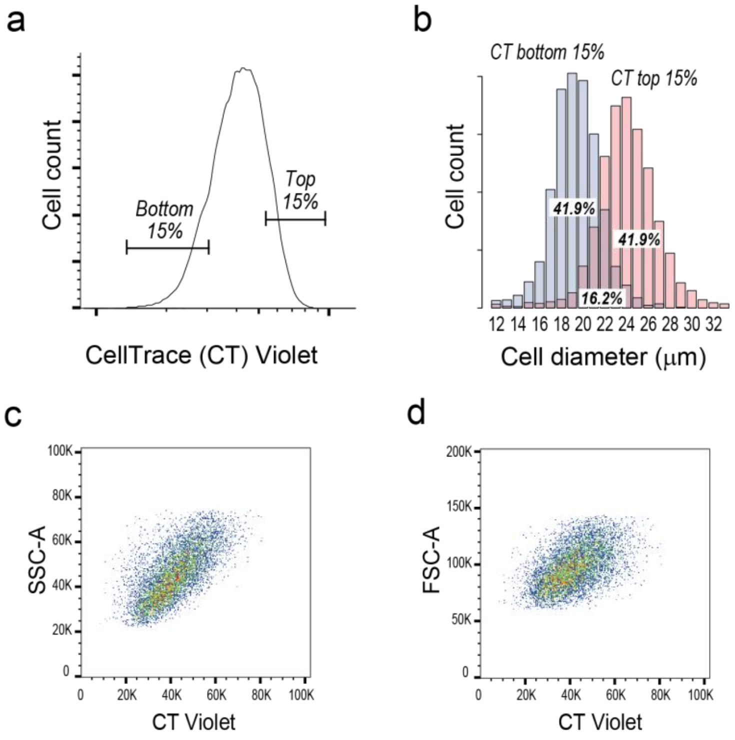

Supplementary Figure 2. Cell size correlation with CT dye intensity. (**a**) Distribution of CT dye intensity of quiescent REF/E23 cells. The top and bottom 15% of the cell population with the highest and lowest CT intensity, respectively, are marked. (**b**) The top and bottom 15% of cells in **a** were isolated by FACS and measured for their cell size distributions based on the Coulter Principle (see Methods). Higher CT intensity was seen correlated with larger cell size. (**c, d**) Positive correlation between CT dye intensity (x-axis) and SSC and FSC measurements (y-axis), respectively. SSC and FSC are generally considered as cell-size indicators.

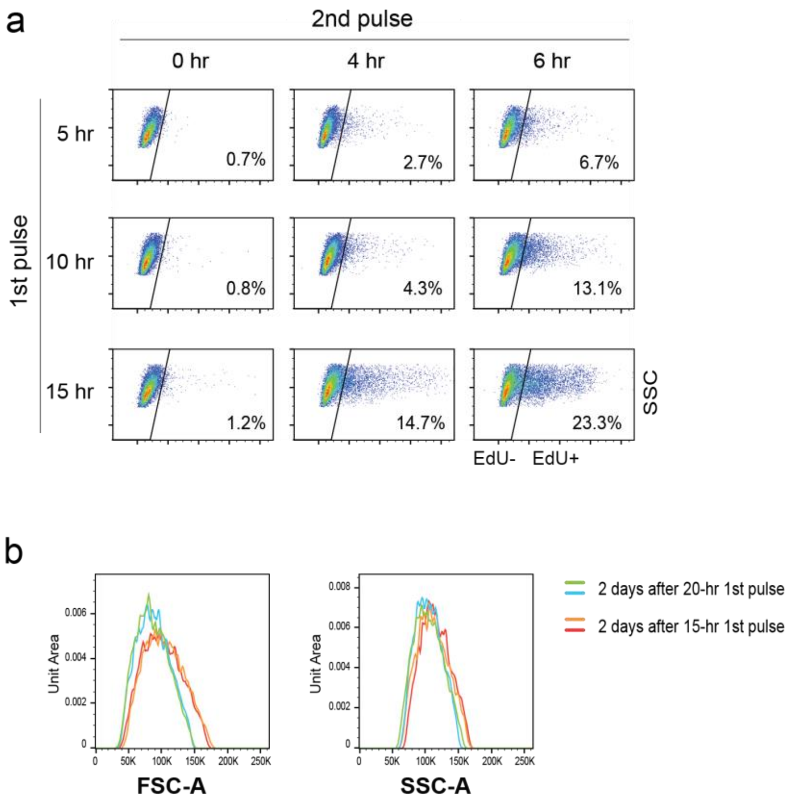

Supplementary Figure 3. Parallel readouts of Fig. 2 experiments. (**a**) EdU assay readout. EdU was added to the culture medium right before the 2nd serum pulse (see Fig. 2a) and kept in the medium until one day after the initiation of the 2nd serum pulse, when cells were harvested for EdU assay. The EdU incorporation result is consistent with the E2F-dGFP readout of Fig. 2c. That is, quiescent cells previously stimulated with the 1st serum pulse of increasing length (with mimosine blockage of G1/S transition) exhibited an increasingly higher response rate (EdU+%) to the 2nd serum pulse. (**b**) FSC and SSC readouts of cell samples in Fig. 2f,g right before the 2nd serum pulse. Quiescent cells previously treated with 20- vs. 15-hr 1st serum pulse had smaller FSC and SSC readings (duplicates), indicating relatively smaller but not larger cell size.

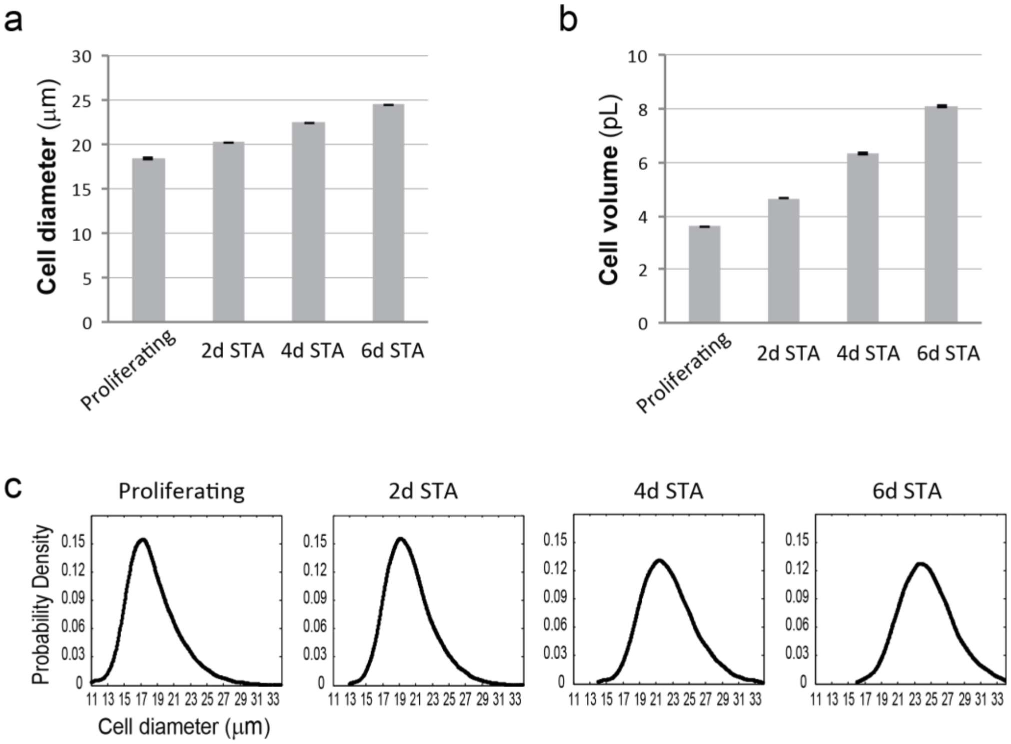

Supplementary Figure 4. Cell size increase of REF/E23 cells under serum starvation. Cell size measurements in diameter (**a**) and volume (**b**) of cycling cells and quiescent cells induced by serum starvation for 2, 4, and 6 days (2d, 4d, 6d STA, respectively). Each column represents the corresponding sample mean ± s.e.m. (20,000-30,000 cells). (**c**) Probability density distributions of cell size (diameter) of cell samples in **a**.

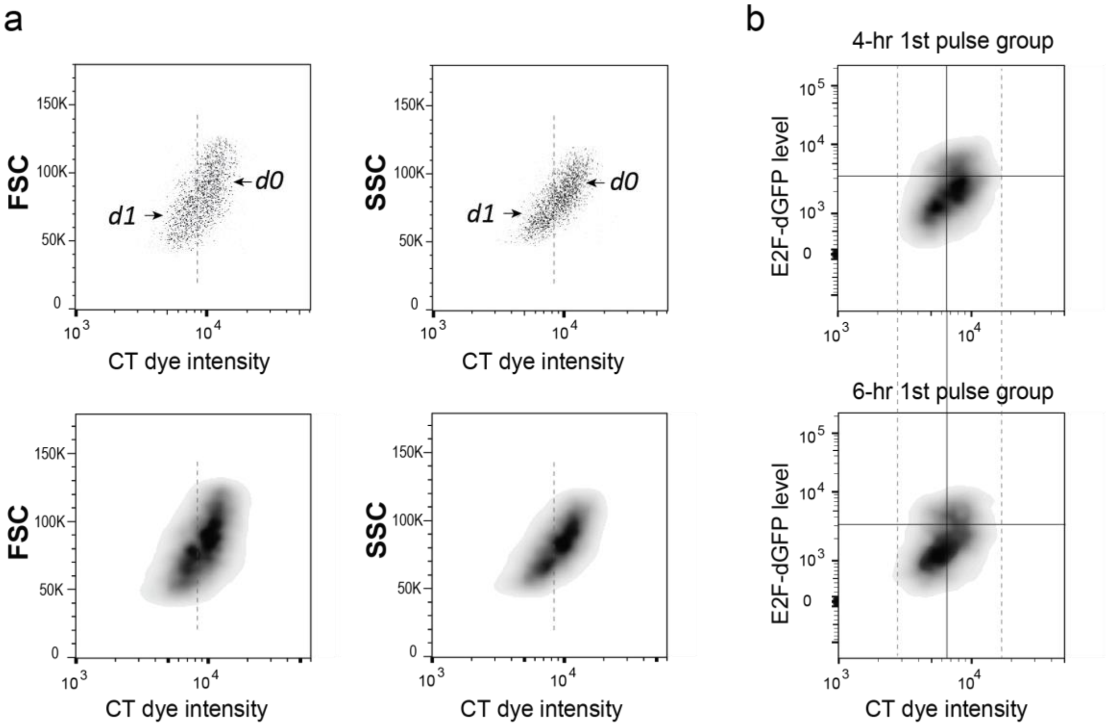

Supplementary Figure 5. Cell size estimate and E2F-ON gating of *d1* and *d0* cells. (**a**) Non-divided (*d0*) and divided (*d1*) cells following the 1st serum pulse (as in Fig. 3a, *pd* = 4 hrs) were measured for their FSC and SSC in flow cytometry right before the 2nd serum pulse. The separation of *d1* and *d0* cells according to CT intensity was the same as in Fig. 3b. The smaller FSC and SSC readings of *d1* vs. *d0* cells indicated their smaller cell size. The upper and lower panels correspond to unsmoothed and smoothed density plots, respectively. (**b**) Smoothed density plot of the flow cytometry result as in Fig. 3c. Solid vertical and horizontal gating lines were placed through the least overlapped regions between cell subpopulations.

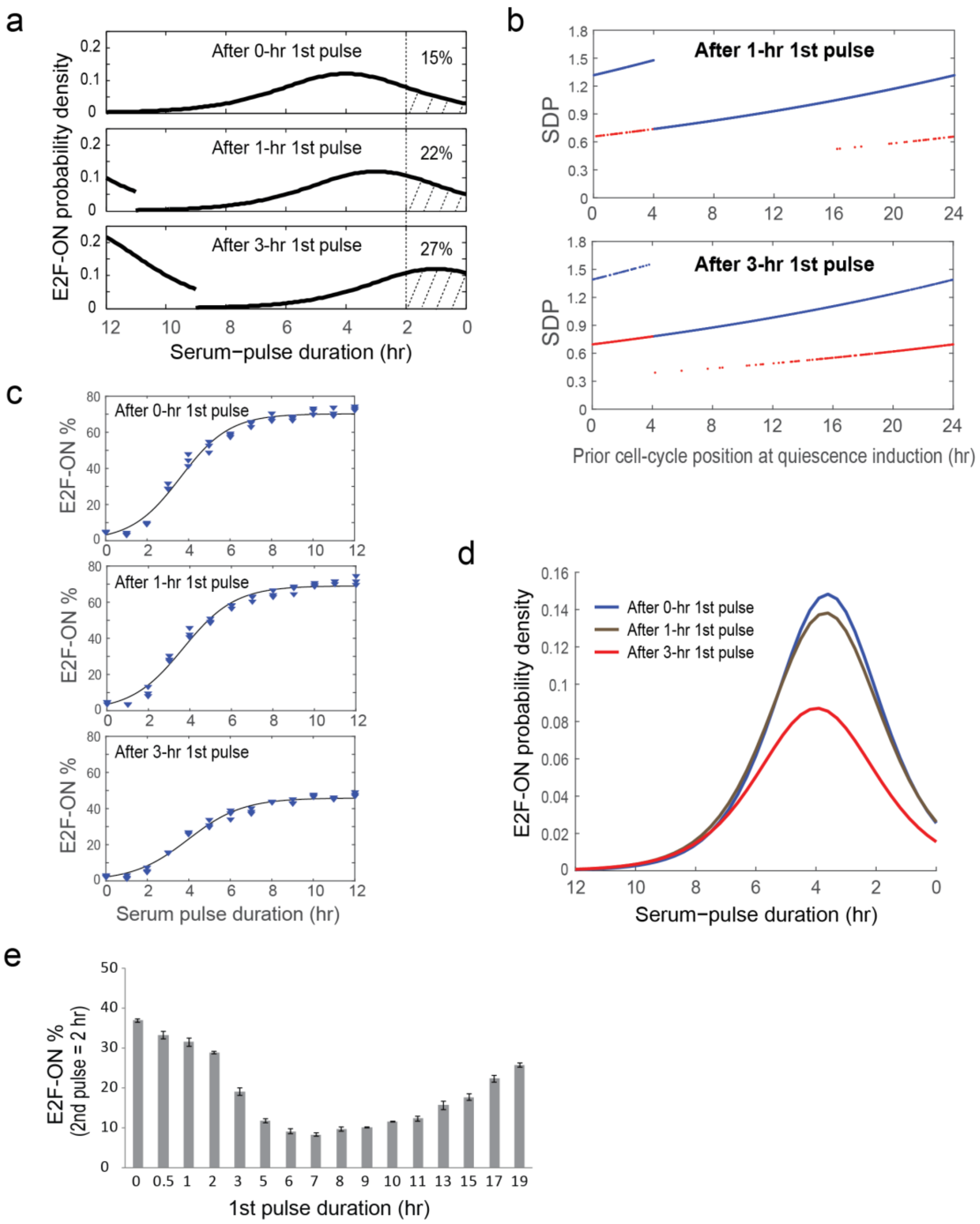

Supplementary Figure 6. Simulation results of competing quiescence models. (**a**) Quiescence-exit distribution after the 1st serum pulse according to the G1 model. Following the 1st pulse of increasing durations (0-3 hrs), more cells enter the cell cycle from the proximal end of the initial distribution (Fig. 1c), which leads to a right shift of the distribution curve and an increasing number of divided cells at the distal tail. Shaded areas indicate the calculated cell fraction (given the accordingly updated distribution in each case) that will enter the cell cycle in response to the 2nd serum pulse (2 hrs). (**b**) In the G0/G1 model, a longer 1st pulse (3- vs. 1-hr) drives more cells to divide without completing the full cycle of serum-dependent *SDP*

accumulation, resulting in a larger portion of quiescent cells with reduced *SDP* (red dots). X-axis indicates the preceding cell cycle position at quiescence induction before the 1st pulse. (**c**) Simulated cumulative quiescence-exit curves based on the updated *SDP* distributions in **b** retain the sigmoidal nature. Each curve indicates a best fit of triplicate data points with the function form as in Fig. 1b. (**d**) Updated quiescence-exit distributions following the 1st serum pulse (according to the G0/G1 model), as derivatives of the sigmoidal curves in **c**. A longer 1st pulse leads to a reduced height of the quiescence-exit distribution. (**e**) E2F-ON% in response to the 2nd serum pulse (*pd* = 2 hrs) following the experimental scheme in Fig. 5a,b. The 1st serum pulse duration is indicated on the x-axis. Each column represents the E2F-ON% (mean ± s.e.m.) calculated from three sets of stochastic simulations (500 runs each).

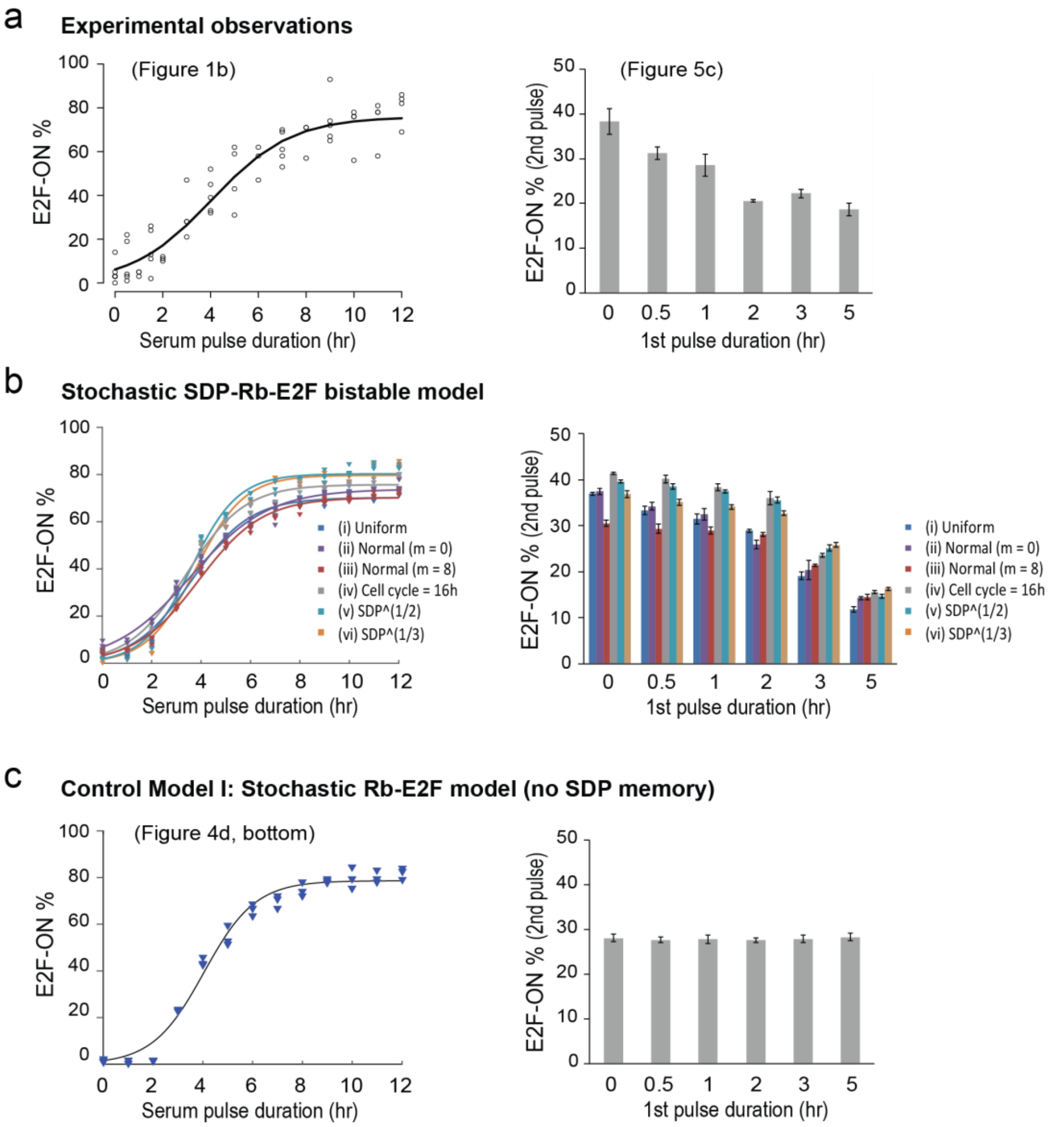

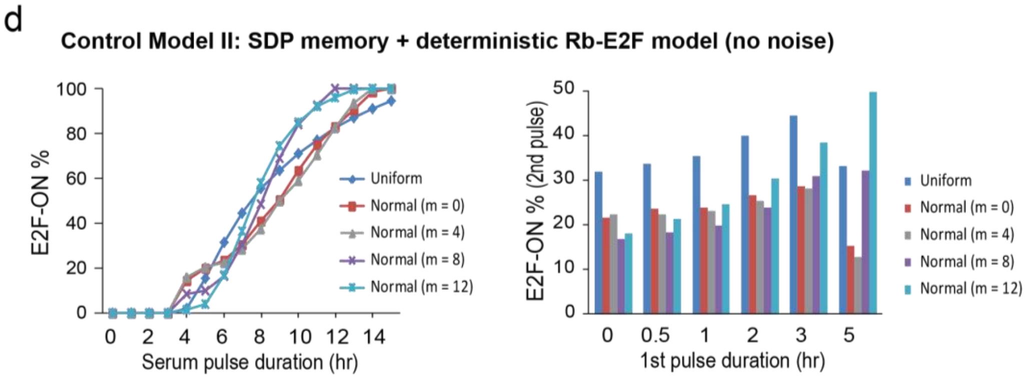

Supplementary Figure 7. Models lacking either SDP memory or Rb-E2F system noise did not reproduce experimental observations. Experimentally observed (**a**) and model simulated (**b-d**) results are shown. Reproduced panels from previous figures are as indicated. (Left) Cumulative quiescence-exit curves. E2F-ON% of serum-starved quiescent cells in response to serum pulses at 20% for indicated durations. Each curve in **a-c** indicates a best fit of data points (with the function form as in Fig. 1b). (Right) E2F-ON% in response to the 2nd serum pulse following the experimental scheme in Fig. 5a,b. The 1st serum pulse duration is indicated (x-axis). Both uniform and normal distributions (with indicated mode positions, *m*) of cells along the cell cycle at quiescence induction were examined in **b** and **d**. Additionally, *SDP*-Rb-E2F models with the cell cycle length = 16 hrs (instead of 24 hrs as in Fig. 4a) and with the serum response elements coupled to the square or cube root of *SDP* (instead of *SDP* itself, Supplementary Table 2) were examined in **b**. Linear batch-scaling factor *Sc* was chosen to match experimental results in **a** when possible (= 1.25 in **b**(i,iv-vi) & **c** and = 1.80 in **d**, for the 2nd pulse; = 1.1 and 1.4 in **b**(ii,iii) for the 1st and 2nd pulse, respectively). In **b**(v,vi), SDP_{c0} = 1.43 (see Methods). The *SDP*-Rb-E2F models (**b**) but not control models (**c**, **d)** reproduced experimentally observed decreasing E2F-ON% following an increasingly longer 1st serum pulse (**a**), with significant negative slopes ($p < 0.05$, in **b**(i-vi)). Error bar in **b** and **c** represents s.e.m. from three sets of stochastic simulations (500 runs each).

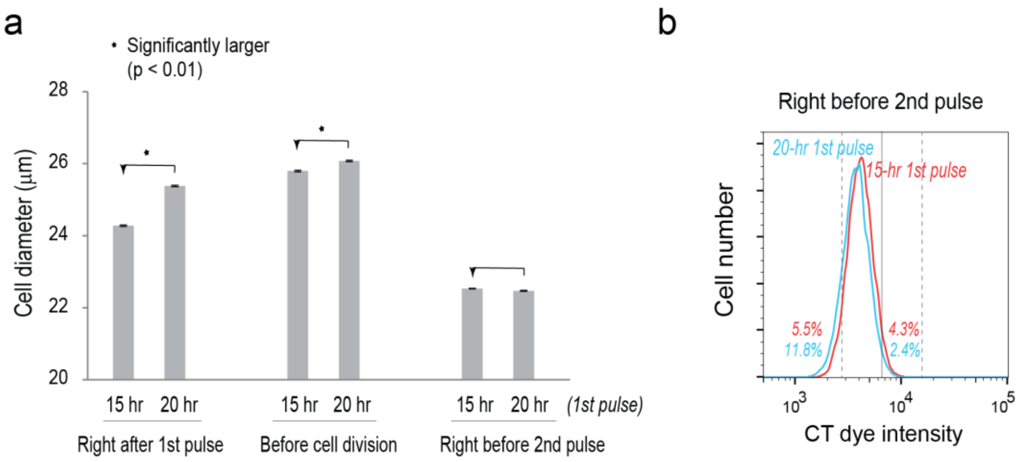

Supplementary Figure 8. Cell size change after 1st serum pulse. The cell size right after the 1st serum pulse (15- and 20-hr) was experimentally measured as in Fig. 2f. After that, cells continued to grow in size under serum starvation until cell division (24 hr), with an intermediate transition rate $V_{t2} = V_{t1}2^{\Delta t * f}$ ($\Delta t = \frac{t2-t1}{24}$, *f = 0.7*), as compared to the original exponential growth rate with serum, $V_{t2} = V_{t1}2^{\Delta t}$. The calculated cell size (in diameter $D \propto \sqrt[3]{V}$, mean ± s.e.m. of 20,000-30,000 cells) at the 24th hr (Before cell division) is shown in the middle part of **a**. After cell division, cells grew at a slower rate as determined in Supplementary Fig. 4a in quiescent cells, $D_{t2} = D_{t1} + \alpha * \Delta t$ ($\alpha = 1.05\ \mu m\ day^{-1}$). In addition, following the 15- vs. 20-hr 1st serum pulse, while the vast majority of cells divided once, a small subset of cells divided twice into even smaller cells (5.5% vs. 11.8% in the cell population as seen before the 2nd pulse, respectively, **b**) and another small subset of cells did not divide and kept large size (4.3% vs. 2.4%, respectively, **b**). The calculated cell size (mean ± s.e.m., after 2 days of growth in quiescence and adjusting for cell division variations) is shown in the right part of **a** (Right before 2nd pulse). Star sign indicates statistical significance ($p < 0.01$) in one-sided (arrow pointed) t-test comparing the sample means. The vertical gating lines in **b** are the same as in Fig. 2h.

Supplementary Table 1. Fractions of quiescent REF/E23 cells responding to serum pulses (20% serum)

**Duration (hr)**	Batch 1	Batch 2	Batch 3	Batch 4	Batch 5	Batch 6	Batch 7	Batch 8	Batch 9	**Mean**	N	SEM
**0.0**	0.03	0.03	0.03	0.03	0.03	0.05	0.03	0.00	0.14	**0.04**	9	0.01
**0.5**					0.03	0.22	0.04	0.01	0.19	**0.10**	5	0.04
**1.0**	0.05	0.03	0.05							**0.04**	3	0.01
**1.5**					0.11	0.26	0.13	0.02	0.24	**0.15**	5	0.04
**2.0**	0.12	0.11	0.10							**0.11**	3	0.01
**3.0**			0.28	0.21		0.47	0.28			**0.31**	4	0.06
**4.0**	0.32		0.33	0.45		0.52	0.39			**0.40**	5	0.04
**5.0**			0.43		0.31	0.62	0.59			**0.49**	4	0.07
**6.0**	0.58		0.47	0.62						**0.56**	3	0.04
**7.0**		0.53	0.58	0.70		0.69	0.61			**0.62**	5	0.03
**8.0**	0.71		0.71		0.57					**0.66**	3	0.05
**9.0**		0.72	0.93		0.65	0.74	0.67			**0.74**	5	0.05
**10.0**						0.76	0.76	0.56	0.78	**0.72**	4	0.05
**11.0**						0.78	0.78	0.58	0.81	**0.74**	4	0.05
**12.0**						0.82	0.84	0.69	0.86	**0.80**	4	0.04

N: sample size;
SEM: standard error of the mean.

Supplementary Table 2. The SDP-Rb-E2F model (adapted from Ref. 1, with changes marked with $\bullet$).

$\frac{d[M]}{dt} = \frac{k_M[S]}{K_S + [S]} \cdot (SDP \cdot S_C)\bullet - d_M[M]$
$\frac{d[E]}{dt} = k_{E0}\bullet + k_E\left(\frac{[M]}{K_M + [M]}\right)\left(\frac{[E]}{K_E + [E]}\right) + \frac{k_b[M]}{K_M + [M]} + \frac{k_{P1}[CD][RE]}{K_{CD} + [RE]} + \frac{k_{P2}[CE][RE]}{K_{CE} + [RE]} - k_{RE}[R][E] - d_E[E]$
$\frac{d[CD]}{dt} = \frac{k_{CD}[M]}{K_M + [M]} + \frac{k_{CDS}[S]}{K_s + [S]} \cdot (SDP \cdot S_C)\bullet - d_{CD}[CD]$
$\frac{d[CE]}{dt} = \frac{k_{CE}[E]}{K_E + [E]} - d_{CE}[CE]$
$\frac{d[R]}{dt} = k_R + \frac{k_{DP}[RP]}{K_{RP} + [RP]} - k_{RE}[R][E] - \frac{k_{P1}[CD][R]}{K_{CD} + [R]} - \frac{k_{P2}[CE][R]}{K_{CE} + [R]} - d_R[R]$
$\frac{d[RP]}{dt} = \frac{k_{P1}[CD][R]}{K_{CD} + [R]} + \frac{k_{P2}[CE][R]}{K_{CE} + [R]} + \frac{k_{P1}[CD][RE]}{K_{CD} + [RE]} + \frac{k_{P2}[CE][RE]}{K_{CE} + [RE]} - \frac{k_{DP}[RP]}{K_{RP} + [RP]} - d_{RP}[RP]$
$\frac{d[RE]}{dt} = k_{RE}[R][E] - \frac{k_{P1}[CD][RE]}{K_{CD} + [RE]} - \frac{k_{P2}[CE][RE]}{K_{CE} + [RE]} - d_{RE}[RE]$

Variables:
S: serum concentration
M: Myc
E: E2F
CD: Cyclin D/Cdk4,6
CE: Cyclin E/Cdk2
R: Rb family proteins
RP: Phosphorylated Rb
RE: Rb-E2F complex

Initial condition:
[*M*] = [*E*] = [*CD*] = [*CE*] = [*RP*] = 0 nM, [*R*] = 2.83 nM$\bullet$, [*RE*] = 0.33 nM$\bullet$.

Note: (**a**) Model parameters are adapted from Ref. 1 and defined in Supplementary Table 3 (including newly added parameters $SDP, S_C,$ and k_{E0}). (**b**) The initial concentrations [*R*] and [*RE*] are determined as steady state values at [*S*] = 0, with the initial concentrations of other variables set at 0 in the model. It fits the expectation that in quiescence, Rb is in excess over E2F, and E2F is present in the Rb-E2F complex instead of free form. (**c**) k_{E0} (basal E2F synthesis rate) is introduced so that the simulated serum threshold and half-activation time to turn on E2F (after newly added parameters and modified initial conditions) are similar to those in the previous Rb-E2F model[1].

Supplementary Table 3. Model parameters (adapted from Ref. 1)

Symbol	Values	Description
k_M	1.0 nM hr^{-1}	Rate constant of Myc synthesis driven by growth factors
k_E	0.4 nM hr^{-1}	Rate constant of E2F synthesis driven by Myc and E2F
k_b	0.003 nM hr^{-1}	Rate constant of E2F synthesis driven by Myc alone
k_{E0}	0.01 nM hr^{-1}	Rate constant of E2F basal/constitutive synthesis
k_{CD}	0.03 nM hr^{-1}	Rate constant of CycD synthesis driven by Myc
k_{CDS}	0.55 nM hr^{-1}	Rate constant of CycD synthesis driven by growth factors
k_{CE}	0.35 nM hr^{-1}	Rate constant of CycE synthesis driven by E2F
k_R	0.18 nM hr^{-1}	Rate constant of Rb constitutive synthesis
k_{P1}	18 hr^{-1}	Phosphorylation rate constant of Rb by CycD/Cdk4,6
k_{P2}	18 hr^{-1}	Phosphorylation rate constant of Rb by CycE/Cdk2
k_{DP}	3.6 nM hr^{-1}	Dephosphorylation rate constant of Rb by phosphatases
k_{RE}	180 nM^{-1} hr^{-1}	Association rate constant of Rb and E2F
K_S	1.2 nM	Michaelis-Menten parameter for CycD and Myc synthesis by growth factors
K_E	0.15 nM	Michaelis-Menten parameter for CycE and E2F synthesis by E2F
K_M	0.15 nM	Michaelis-Menten parameter for CycD and E2F synthesis by Myc
K_{RP}	0.01 nM	Michaelis-Menten parameter for Rb dephosphorylation
K_{CD}	0.92 nM	Michaelis-Menten parameter for Rb phosphorylation by CycD/Cdk4,6
K_{CE}	0.92 nM	Michaelis-Menten parameter for Rb phosphorylation by CycE/Cdk2
d_M	0.7 hr^{-1}	Degradation rate constant of Myc
d_E	0.25 hr^{-1}	Degradation rate constant of E2F
d_{CD}	1.5 hr^{-1}	Degradation rate constant of CycD
d_{CE}	1 hr^{-1}	Degradation rate constant of CycE
d_R	0.06 hr^{-1}	Degradation rate constant of Rb
d_{RP}	0.06 hr^{-1}	Degradation rate constant of phosphorylated Rb
d_{RE}	0.03 hr^{-1}	Degradation rate constant of Rb-E2F complex
SDP	SDP value of a quiescent cell (calculated as described in Methods)	
S_c	Linear scaling factor to reflect batch variations of baseline serum response in different experiments ($S_c = 1.0$, unless otherwise noted).	

Supplementary Discussion

It is interesting why in Fig. 2 experiments larger cells at quiescence induction (following the 1st serum pulse) were not larger anymore by the time of starting the 2nd serum pulse. As seen in Fig. 2e, with the time separation between the starts of the 1st and 2nd pulses being the same (3 days), cells with the 15- vs. 20-hr 1st pulse underwent a 5-hr shorter serum exposure but meanwhile a 5-hr longer serum starvation period. If cells continued to increase size under serum starvation, they would make up (at least partially) the size difference caused by shorter 1st pulse. Indeed, under serum starvation, REF/E23 cells continued to increase size (Supplementary Fig. 4, although with a slower rate than the exponential growth within the cell cycle, Fig. 4a), consistent with high metabolic activities of quiescent fibroblasts[2]. By assuming an intermediate transitional growth rate under serum starvation before exiting the cell cycle, and adjusting for the small cell fractions that did not divide exactly once following the 1st pulse (Supplementary Fig. 8b), the calculated cell size can qualitatively reproduce the experimental observations (i.e., larger cells at quiescent induction were not larger anymore by the time of 2nd pulse, Supplementary Fig. 8a). Considering the small cell fractions that did not divide exactly once following the 1st pulse did not change the qualitative simulation results in Fig. 4f (i.e., quiescent cells following a 20- vs. 15-hr 1st pulse exhibited a larger response rate to the 2nd pulse).

Similar explanations apply to the experiment in Fig. 2a, except for the involvement of two opposing effects: cells in late G1 likely took longer time to exit the cell cycle under serum starvation (by reverting the G0 → G1 path as G1/S transition was blocked by mimosine), so they had longer in-cell-cycle growth; meanwhile, they were under longer term of effective drug blockage which may negatively affect cell size. Net result of these opposing effects may explain the biphasic cell size change observed by the time of the 2nd pulse (Fig. 2b).

Supplementary References

1. Yao, G., Lee, T.J., Mori, S., Nevins, J.R. & You, L. A bistable Rb-E2F switch underlies the restriction point. *Nat Cell Biol* **10**, 476-82 (2008).
2. Lemons, J.M.S. et al. Quiescent Fibroblasts Exhibit High Metabolic Activity. *PLoS Biol* **8**, e1000514 (2010).
